# Genetic counseling in times of genomic analyses – current aspects of common topics in human genetics practice

**DOI:** 10.1515/medgen-2021-2050

**Published:** 2021-05-14

**Authors:** Susanne Morlot, Christian Netzer

**Affiliations:** Institute of Human Genetics, Medizinische Hochschule Hannover; OE 6300, Carl Neuberg Str. 1, 30625Hannover, Germany; Institute of Human Genetics, University of Cologne University Hospital of Cologne, Kerpener Str. 34, 50931Cologne, Germany

There is an almost exponential increase in requests for genetic counseling, and the expectations of those seeking advice have also risen. Genetic testing has become more and more important in several respects: it can clarify the hereditary nature of a disease and the risk of recurrence for family members. It prepares the ground for prenatal and preimplantation genetic diagnostics. And by establishing a precise diagnosis, it can resolve whether using an innovative and specific therapeutic approach is an option. The geneticist has to be (at least on a superficial level) a generalist for almost all other medical specialties. Increasingly, decisions have to be made for difficult questions: Which genetic test is medically indicated – and how extensive should this test be, also regarding legal aspects? The new Uniform Value Scale (*Einheitlicher Bewertungsmaßstab*, EBM) provides us with the theoretical option to sequence the exome or even genome in any patient with a rare disease. Which diagnostic tests (possibly in which order) are best suited to provide helpful information – also taking into account the costs of the diagnostic test in relation to its possible benefit?

This issue of *medizinische**genetik***, therefore, focuses on three topics:

– How can the growing demand for genetic counseling be managed (keywords “counseling assistant”, telemedicine)?
– How can a good quality of genetic counseling be guaranteed despite increasing demands?
– Which genetic tests should be offered for frequent questions (e. g. positive family history for autosomal recessive disorders, abortions, reproductive medicine)?

The article by **Christian Schaaf** “Genetic Counseling in the United States” uses the example of the United States to show very clearly how the importance of and need for genetic counseling has increased internationally in recent years. He describes the possible deployment of *genetic counselors* in the field of human genetics that has already been established in the United States: “Genetic counselors represent an indispensable, well-established and well-integrated group of healthcare providers in the field of genetic and genomic medicine”.

Following the examples of the USA and European countries, a training course for the “Genetic Counsellor Profession” has been established at the University of Innsbruck under the leadership of Johannes Zschocke. Based on the results of a workshop “Prospects and Challenges for the Genetic Counsellor Profession in the German-Speaking Countries: Report of a Workshop”, **Gunda Schwaninger et al.** report on the contents, possibilities and problems of legal and billing regulations of this newly established profession in German-speaking countries.

In the future, telemedicine will make a significant contribution to improving care in Germany, especially in structurally weak regions. Although telemedicine has already been well established in many model projects, neither the legal nor the billing problems that genetic counseling faces have yet been sufficiently addressed. The extensively researched article by **Johanna Tecklenburg** “Telemedicine – Chances and Challenges for Medical Genetics in Germany” sheds light on this topic in more detail.

“For carrying out a quality management of genetic counseling it is important to know, what it means and what the tenors are”. In his article, **Friedmar Kreuz’** introductory sentence summarizes what has to be taken into account in genetic counseling besides a good professional communication of content: What is it that the person seeking advice wants to know, where does the focus need to be set, and what does it mean for the family? Many points in the article “Genetic counseling: How to carry out a quality management” entice thoughtful reflection on one’s own counseling style.

Carrier *tests* for autosomal recessive disorders have been offered for 30 years whenever there is a recognizable increased risk for a certain disease in a family. In the article co-authored by one of us (**Christian Netzer et al.**), the practice that has resulted from this is presented with its particularities and pitfalls. Almost inevitably, this raises the question of whether such a strategy is still up-to-date in times where comprehensive (or “expanded”) carrier *screening* is technically feasible.

10–15 % of all couples are involuntarily childless, and in a significant proportion of cases this is due to genetic causes. The article by **Margot Wyrwoll et al.** “Genetic counseling and diagnostic guidelines for couples with infertility and/or recurrent miscarriage” addresses the implications of these genetic causes of male and female infertility, which complement the most common cause of infertility – increasing maternal age. A precise algorithm for the diagnostic workup of affected couples completes the article.

**Johannes Zschocke** presents in his article the recently developed International Classification of Inherited Metabolic Disorders (ICIMD). In this classification, the predominantly monogenic, genetically already well-defined metabolic diseases are assigned to the respective clinical and biochemical phenotypes in 8 disorder categories and 24 subgroups. The results represent a work in progress, in which genes not yet assigned to a specific phenotype or function can easily be matched in the course.

In summary, this special issue is intended to provide an overview of the “state of the art” in common genetic questions and the status of complementary options to cope with the increasing number of consultation requests. We sincerely thank all the dedicated authors, coauthors, reviewers, and organizers of this issue who contributed to its success.

